# Can active components of licorice, glycyrrhizin and glycyrrhetinic acid, lick rheumatoid arthritis?

**DOI:** 10.18632/oncotarget.6200

**Published:** 2015-10-20

**Authors:** Qing-Chun Huang, Mao-Jie Wang, Xiu-Min Chen, Wan-Lin Yu, Yong-Liang Chu, Xiao-Hong He, Run-Yue Huang

**Affiliations:** ^1^ Department of Rheumatology, The Second Affiliated Hospital, Guangzhou University of Chinese Medicine (Guangdong Provincial Hospital of Chinese Medicine), Guangzhou, China; ^2^ Central Laboratory, The Second Affiliated Hospital, Guangzhou University of Chinese Medicine (Guangdong Provincial Hospital of Chinese Medicine), Guangzhou, China

**Keywords:** licorice, glycyrrhizin, glycyrrhetinic acid, rheumatoid arthritis, the COX-2/TxA2 pathway

## Abstract

**OBJECTIVES:**

This review stated the possible application of the active components of licorice, glycyrrhizin (GL) and glycyrrhetinic acid (GA), in rheumatoid arthritis (RA) treatment based on the cyclooxygenase (COX)-2/thromboxane A2 (TxA2) pathway.

**METHODS:**

The extensive literature from inception to July 2015 was searched in PubMed central, and relevant reports were identified according to the purpose of this study.

**RESULTS:**

The active components of licorice GL and GA exert the potential anti-inflammatory effects through, at least in part, suppressing COX-2 and its downstream product TxA2. Additionally, the COX-2/TxA2 pathway, an auto-regulatory feedback loop, has been recently found to be a crucial mechanism underlying the pathogenesis of RA. However, TxA2 is neither the pharmacological target of non-steroidal anti-inflammatory drugs (NSAIDs) nor the target of disease modifying anti-rheumatic drugs (DMARDs), and the limitations and side effects of those drugs may be, at least in part, attributable to lack of the effects on the COX-2/TxA2 pathway. Therefore, GL and GA capable of targeting this pathway hold the potential as a novel add-on therapy in therapeutic strategy, which is supported by several bench experiments.

**CONCLUSIONS:**

The active components of licorice, GL and GA, could not only potentiate the therapeutic effects but also decrease the adverse effects of NSAIDs or DMARDs through suppressing the COX-2/TxA2 pathway during treatment course of RA.

## INTRODUCTION

**Figure 1:**
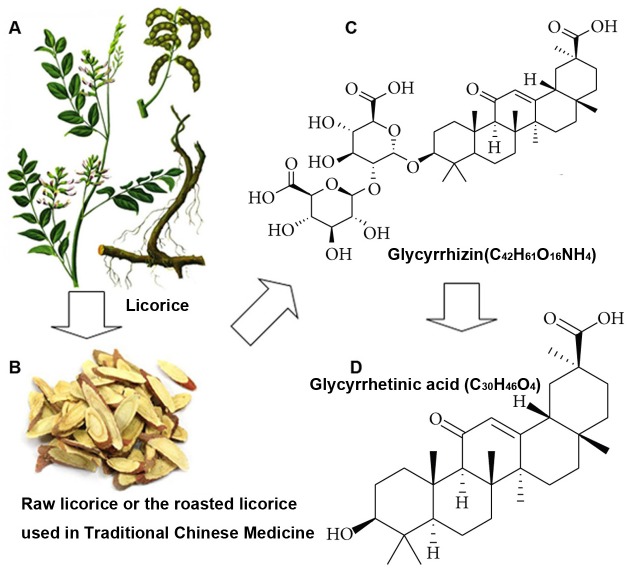
Licorice and its major active components **A.**, the Figure of licorice herb is selected from The Compendium of Materia Medica (Bencao Gangmu). **B.**, raw licorice and the roasted licorice are frequently employed as medications in traditional Chinese Medicine. **C.** and **D.**, the chemical structures of glycyrrhizin (GL) and glycyrrhetinic acid (GA).

Because of the unwanted side effects of current drugs used for rheumatoid arthritis (RA) treatment, botanical medicines have become popular as alternative remedies as they are believed to be efficacious, safe and have over a thousand years’ experience in treating patients [1]. In addition, analysis of patents on anti-RA therapies issued in China revealed that traditional Chinese Medicine may provide substantial new information for anti-RA drugs development [2]. Licorice (Glycyrrhiza glabra) is a well-known plant, which is utilized to add flavor to foods, beverages, and tobacco, and it is also used as a medicinal plant [3]. The principle component of licorice is Glycyrrhizic acid or glycyrrhizin (GL), which is a natural and major pentacyclic triterpenoid glycoside of licorice roots extracts [4] (Figure 1). GL is readily hydrolyzed to glycyrrhetinic acid (GA) in human body [5]. Following oral administration in humans as well as in rats, GL is metabolized in the gastrointestinal tract by glucuronidases into GA, which can be totally absorbed [6].

Licorice remains one of the most prescribed herbs in Chinese Medicine. There is much literature on the biological effects of the major bioactive components of licorice, particularly in terms of their anti-cancer, anti-inflammatory and anti-arthritic effects [1, 5, 7]. For example, licorice and the roasted licorice have benefits in protecting against both acute inflammation and chronic inflammatory conditions including RA [7].

It is well known that cyclooxygenase (COX)-2 is an important target of licorice, as many constituents of licorice are able to suppress COX-2 [1, 8, 9], which is critically involved in the pathogenesis of tumor and inflammatory diseases like RA [10-13]. Five years ago, Prof. Paul M. Stewart and Stephen M. Prescott raised a question, that is, can licorice lick colon cancer?[14]. This question is raised from a discovery showing that GL reduced COX-2 activity, tumor growth, and metastasis, without the adverse effects associated with non-steroidal anti-inflammatory drugs (NSAIDs) and selective COX-2 inhibitors (COXIBs) [15]. Today, using the same sentence pattern, we are asking the question “can active components of licorice GL and GA lick RA?” This is a data-based question, raised from several lines of evidence showing as followings. First, GL and GA provide an anti-inflammatory effect by suppressing the expression and activity of COX-2 through the inhibition of nuclear factor (NF)-κB and phosphoinositide-3-kinase (PI3K) activity [16]. Second, there are some common targets and common therapies have been revealed between RA and cancers, such as cadherin-11 and COX-2 [11, 17]. Third, COX-2 is crucially implicated in RA pathogenesis, and NSAIDs as well as COXIBs are frequently used in treating patients with RA [18]. Therefore, can active components of licorice GL and GA lick RA? In this review, we will clarify this possibility based on the COX-2/thromboxane A2 (TxA2) pathway, which is a mechanism novelly delineated in the pathogenesis of RA [11, 19].

## METHODS

We searched the PubMed database from inception to July 2015 with the following search terms: “licorice”, “glycyrrhiza glabra”, “glycyrrhizic acid”, “glycyrrhizin”, “glycyrrhetinic acid”, “thromboxane”, “cyclooxygenase-2”, and/or “rheumatoid arthritis”. The references within the selected reports were also considered. No limitations on language and study types. Relevant literature focusing on the field of licorice and its active components, as well as RA was identified. Three independent investigators conducted the searching process, and the experts in the field of Rheumatology were involved in the procedure of literature analysis.

## THE COX-2/TXA2 PATHWAY IS A PHARMACOLOGICAL TARGET OF GL AND GA

**Figure 2:**
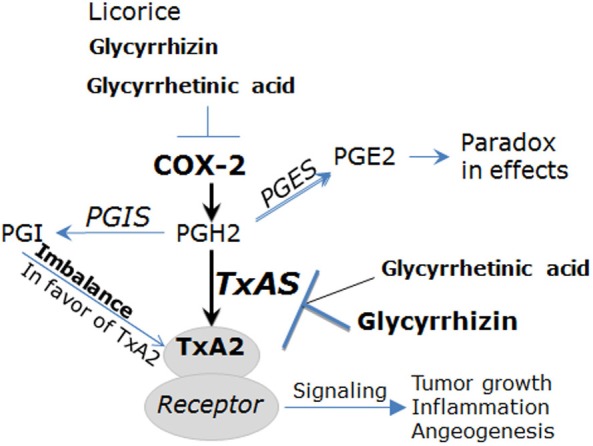
The COX/TxA2 pathway is the pharmacological target of glycyrrhizin (GL) and glycyrrhetinic acid (GA) Among downstream products of COX-2 pathway, PGI is generally considered to have cytoprotection effects, and the imbalance of PGI/TxA2 in favor of the latter is one of critical mechanisms underlying pathogenesis of cancer, inflammatory disease and vascular disorders. The role of PGES/PGE2 is to some extent controversial, as PGE2 has both pro-inflammatory and immunosuppression effects depending on cell context. TxA2 acts through binding with TxA2 receptor (TP), thereby exerting promoting effects for tumor growth, inflammation and angiogenesis. Licorice and its active components GL and GA are considered to hold anti-inflammatory and anti-cancer properties through targeting the COX-2/TxA2 pathway. For example, GA inhibits lung tumor growth through suppressing expression and activity of COX-2 and TxAS and the downstream ERK/CREB signaling (Ref.^5^). Abbreviations: COX-2, cyclooxygenase-2; PGH2, prostaglandin H2; PGE2, prostaglandin E2; PGI, prostacyclin; TxA2, thromboxane A2; PGES, prostaglandin E synthase; PGIS, prostacyclin synthase; TxAS, thromboxane A2 synthase.

It is well known that COX-2 is an inducible enzyme becoming abundant in inflammatory diseases including RA [11, 20]. COX-2 catalyzes the conversion of arachidonic acid (AA) into prostaglandin H2 (PGH2). PGH2 is unstable and it is catalyzed by prostaglandin E synthase (PGES), prostacyclin synthase (PGIS) and thromboxane synthase (TxAS) into prostaglandin E2 (PGE2), prostacyclin (PGI) and TXA2, respectively [21, 22] (Figure 2). The role of PGES/PGE2 is to some extent controversial, as PGE2 has both pro-inflammatory and immunosuppression effects depending on cell context [22]. PGIS is generally considered to have cytoprotection effects, and the imbalance of PGI/TxA2 in favor of the latter is one of critical mechanisms underlying pathogenesis of cancer, inflammatory disease and vascular disorders [21, 22] (Figure 2). As a downstream product of COX-2 in inflammatory sites, TxA2 is a local hormone acting close to the site of its synthesis via autocrine or paracrine manner [19, 21, 22]. TxA2 functions through binding with its signature receptor (Figure 2), TxA2 receptor (TP), which is a member of the G-protein-coupled cell surface receptor family [11, 22, 23]. Importantly, it is now clear that the COX-2/TxA2 pathway is an auto-regulatory feedback loop for biosynthesis and action of TxA2 and it plays a key role in the pathogenesis of RA [11, 19].

Early studies documented that as the active component of licorice, GL is an inhibitor of COXs, thereby having anti-inflammatory and anti-tumor effects [24, 25]. Subsequently, GL was shown to have protective effects on acute liver injury via downregulation of proinflammatory mediators including COX-2 [26]. Further study showed that GL potently protected against LPS-induced acute lung injury through, at least in part, the suppression of COX-2 [27]. These studies suggest that GL provides anti-inflammatory effects with low toxicity or cytoprotective property. Several studies revealed that GL is an inhibitor of high mobility group protein B1 (HMGB1) [28], which is known to induce inflammation by enhancement of proinflammatory molecules signaling including COX-2 pathway [29]. Intriguingly, HMGB1 is expected to be a new target for RA treatment [30]. In Wister rats model of 2-acetylaminofluorene (2-AAF)-induced liver toxicity, pretreatment with GA showed potential hepatoprotective effects, which are partly attributable to the attenuation of COX-2 and its transcriptional factor NF-κB [31]. In human endothelial cells, the effects of TP agonist I-BOP could be mimicked by 1μM of GA with a similar time course and efficacy [32], suggesting that GA may exert its biological effects through acting on TxA2 pathway. In our laboratory, it has been found that anti-tumor effect of GL is, at least in part, TxAS-dependent [4]. Additionally, we have elucidated that, through inhibiting TxAS and its initiated extracellular signal-regulated kinas (ERK) / cAMP response element-binding protein (CREB) signaling, GA suppresses lung tumor cell proliferation [5]. It should be noted that the activities of several key molecules COX-2, TxAS and NF-κB were inhibited by GA, the whole effects of COX-2/TXA2 pathway is therefore suppressed by GA. Although GA may function as a TP agonist in some models, theoretically, its effects cannot be mediated through the downstream signalings, as ERK/CREB and NF-κB activities can be significantly inhibited by GA.

Altogether, these observations have revealed that the COX-2/TxA2 pathway is a pharmacological target of GL/GA, which provides new insights into the mechanisms of action of licorice. It also provides an explanation for the anti-inflammatory and anti-tumor effects of GL and GA, as the COX-2/TxA2 pathway is well-known to be the important molecular mechanisms underlying pathogenesis of tumor and RA [11, 19, 20, 33, 34].

## ROLE OF THE COX-2/TXA2 PATHWAY IN THE PATHOGENESIS OF RA

**Figure 3:**
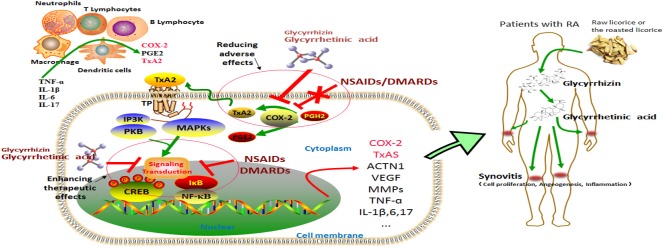
The COX-2/TxA2 pathway is a crucial mechanism underlying the toxicity reducing and efficacy enhancing effects of Glycyrrhizin (GL) and glycyrrhetinic acid (GA) to NSAIDs/DMARDs We have previously elucidated a positive feedback loop for the biosynthesis and action of TxA2, i.e. the COX-2/TxA2 pathway (Ref.^17^). Briefly, in the inflammatory microenvironment of RA joints, lymphocytes and inflammatory cells like macrophages and neutrophils are recruited and produce many pro-inflammatory cytokines, such as TNF-α, IL-1β, IL-6, and IL-17 etc. By stimulation with these cytokines, the crucial molecules of COX-2 pathway, such as PGE2 and TxA2, were produced by those inflammatory cells and RA FLS. Through autocrine or paracrine signaling, TxA2 is able to specifically bind with its signature receptor TP, thereby activating several intracellular signals, MAPKs and PI3K/PKB pathways for instance. The transcription factor CREB and NF-κB can be subsequently activated, and thus inducing the expression of COX-2, TxAS, ACTN1, VEGF and other inflammatory cytokines. Therefore, a positive auto-regulatory feedback loop for the synthesis and action of TxA2 in inflammatory sites is formed and contributes to synovitis, a key role in pathogenesis of RA. It is thus suggested that the pharmacological approaches targeting COX-2/TxA2 pathway hold the potential as a novel add-on therapy in the treatments of RA. Both NSAIDs (including COXIBs) and DMARDs are typically prescribed medications for treatments of patients with RA. TxA2 is believed to be the non-target of NSAIDs and DMARDs. Importantly, the limitations and adverse effects of those drugs may be, at least in part, due to lack of the effects on the COX-2/TxA2 pathway. GL and GA are the major active components of licorice. Following oral administration in humans, GL is metabolized into GA in the gastrointestinal tract and GA can be totally absorbed. Fortunately, GL and GA have been reported to target the COX-2/TxA2 pathway. Therefore, GL or GA could be used as an adjunctive agent in RA treatments not only to enhance the therapeutic effects of NSAIDs/DMARDs but also to reduce the adverse effects associated with NSAIDs/DMARDs. Abbreviations: TNF, tumor necrosis factor; IL, interleukin; RA FLS, rheumatoid arthritis fibroblast-like synoviocytes; COX, cyclooxygenase; TxA2, thromboxane A2; TP, thromboxane A2 receptor; PGH2, prostaglandin H2; PGE2, prostaglandin E2; NSAIDs, non-steroidal anti-inflammatory drugs; DMARDs, disease modifying anti-rheumatic drugs; NF-κB, nuclear factor κB; CREB, cAMP response element-binding protein; MAPKs, mitogen activated protein kinases; PI3K, phosphoinositide-3-kinase; VEGF, vascular endothelial growth factor; ACTN1, α-actinin-1; MMPs, matrix metalloproteinase.

It is well known that TxA2 is one of the downstream products of COX-2, and COX-2 as well as TxA2 are overexpressed in inflammatory conditions like RA [11, 19]. In early studies, the higher TxA2 levels were found in synovial lining obtained from RA patients, as compared to specimens from non-RA patients [35-37]. Moreover, TxA2 release was increased in peripheral blood leucocytes when cultured with RA synovial fluid exudates [38, 39]. Recently, a study recruited 54 RA patients as well as 20 healthy subjects and found that the biosynthesis of TxA2 in RA patients was significantly higher than healthy controls [40], which is in agreement with our recent report for the first time showing that serum level of TxA2 is positively correlated with 28-joint disease activity (DAS28) score of patients with RA [19]. Additionally, we also found that in RA fibroblast-like synoviocytes (RA-FLS), COX-2 effects can be mainly mediated by TxA2, and the mRNA expression of COX-2 is regulated by TxA2 action [11]. This study suggests a positive feedback for TxA2 synthesis and action in RA synovial tissue. Interestingly, it is now clear that in tumor cells, TxA2 contributes to cell proliferation through an auto-regulatory feedback loop, in which NF-κB and its downstream COX-2 are involved [20]. Therefore, enlightened by these findings, we recently determined this pathway in in-vitro model of RA. The results confirmed the existence of an auto-regulatory feedback loop for TxA2 in RA FLS [19]. Through this auto-regulatory feedback loop, transcription factor NF-κB is activated by TxA2 signaling, COX-2 and other inflammatory factors like TNF-α and IL-1 are increased thereafter, thereby contributing to inflammation in RA [19] (Figure 3).

Collectively, these observations suggest that the pharmacological approaches targeting the COX-2/TxA2 pathway hold the potential as a novel add-on therapy in the treatments of RA.

## ADVERSE EFFECTS OF RA TREATMENT DRUGS ARE RELATED TO THE COX-2/TXA2 PATHWAY

Conventional disease-modifying anti-rheumatic drugs (cDMARDs) are the first-line medications used for RA treatment [41], while cDMARDs are more or less ineffective in the late phase of RA and the unwanted side effects often limit their use [42]. For example, both methotrexate (MTX) and leflunomide (LEF) are most frequently prescribed cDMARDs, while use of these two drugs entails a risk of cytopenias and the toxicity of liver and renal [41, 43]. Biologic disease-modifying anti-rheumatic drugs (bDMARDs) generally carry a definite increased infection and cancer risks [41, 44, 45].

It is now clear that, in patients with RA, TxA2 is not the molecular target of DMARDs [19]. The limitations and side effects of these drugs are considered to be, at least in part, due to lack of the effects on the biosynthesis of COX-2-derived TxA2 [11]. In support of this view, it is found that treatment of RA patients with anti-TNF-α agents, belonging to bDMARDs, may blunt isoprostane generation in the absence of significant effects on TxA2 biosynthesis, which could be associated with a higher frequency of non-melanoma skin cancer in patients long-term treated with anti-TNF-α agents [11, 40]. In addition, MTX is found not to suppress TxA2 biosynthesis in whole blood from RA patients although MTX is a preferential COX-2 inhibitor [46]. Interesting, the combined usage of MTX and aspirin (selective TxA2 inhibitor) results in antagonism of the cytotoxic effects of MTX [47]. Therefore, these observations suggest that suppressing the biosynthesis of TxA2 that largely derived from COX-2 in inflammatory sites may produce a strong antagonistic effect to reduce the adverse effects of DMARDs.

Nonsteroidal anti-inflammatory drugs (NSAIDs) including COXIBs are routinely used for long-term therapy of RA in clinical practice [48, 49]. However, because of the action that inhibition of COX-2-derived endothelial PGI2 without concomitant inhibition of TxA2 [50-52], some adverse effects of COXIBs, such as cardiovascular effects and renal effects are to some extent unavoidable [53-59]. Hence, the inhibitors of targeting COX-2-derived TxA2 can theoretically mitigate those adverse effects, when administered in combination with COX-2 inhibitors.

## GL AND GA MAY BE USED AS THE ADDITIVE TO RA TREATMENT AS THEY SUPPRESS THE COX-2/TXA2 PATHWAY

Coupled with the observations showing the positive role of COX-2/TxA2 pathway in pathogenesis of RA, as stated above, the fact that TxA2 is not the molecular target of DMARDs and NSAIDs/COXIBs suggest that drugs targeting COX-2-derived TxA2 may reduce the negative side effects during the RA treatment course. In support of this conclusion, we have previously demonstrated that GL and GA capable of inhibiting COX-2 expression and TxA2 biosynthesis could be used as an adjunctive agent in lung tumor not only to enhance the chemotherapeutic effects of cisplatin but also to reduce the adverse effects associated with cisplatin [4, 5]. In a word, GL or GA can be used as an additive to treatments of RA, as they are able to target the COX-2/TxA2 pathway.

As a matter of fact, many evidences have already documented that GL or GA can be used as an adjunctive agent in treatments of RA. Triptolide, a major active component of Tripterygium wilfordii, is used for treatment in animal models of RA, whereas this natural component possesses various pharmacological activities with narrow therapeutic window and severe toxicities. In animal model studies, toxicity of triptolide can be attenuated with concomitant use of GL, as pretreatment with GL significantly accelerates the metabolic elimination of triptolide from the animal body [60]. Moreover, combined triptolide and GL treatment (triptolide 13.40 μg, GL 26.78 mg) can reduce the arthritic index of collagen induced arthritis (CIA) rats and decrease serum levels of TNF-α, and such effect was similar to the one measured upon application of triptolide 17.86 μg [61]. It is suggested that GL can enhance the chemotherapeutic effects of triptolide. Furthermore, it has been shown that GA, MTX, and combination of GA and MTX (GA+MTX) suppressed the expression of TNF-α and IL-1β in fibroblast-like synovial (FLS) cells from CIA rats in a time-dependent manner, and the suppressing effect is GA+MTX>MTX>GA [62].

**Figure 4:**
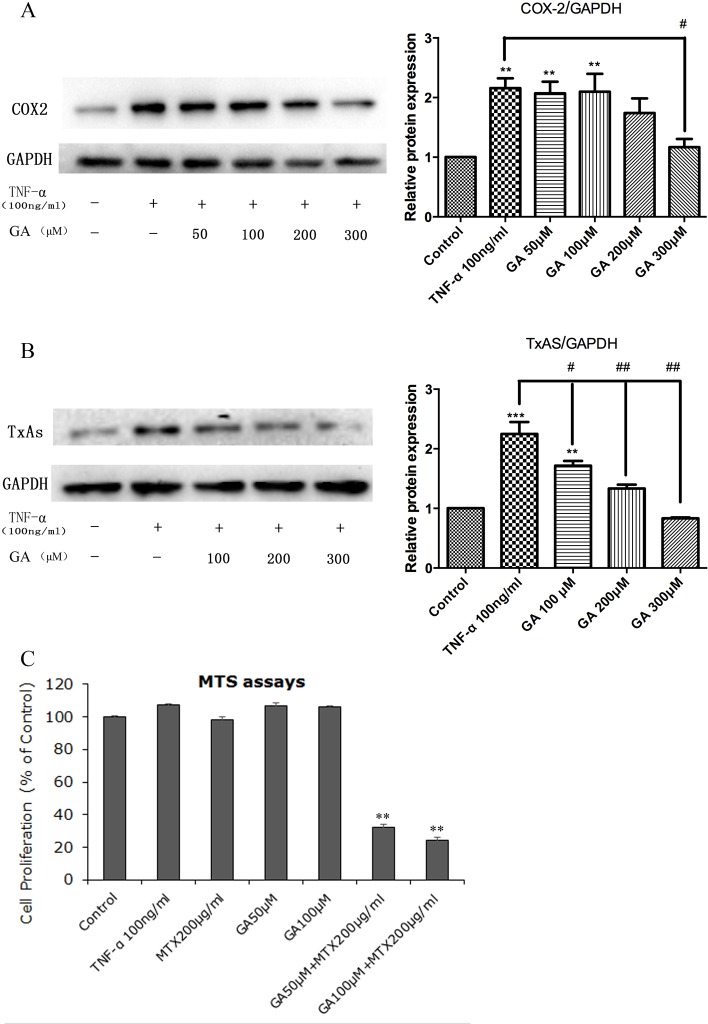
Effects of GA in RA FLS Cells of RA FLS was stimulated with 100 ng/μl TNF-α for 6h, followed by treatment of cells with graded levels of GA for 24h. Cells without treatment served as controls. **A.** and **B.**, GA suppressed COX-2 and TxAS expression in a dose-dependent manner. The protein levels of COX-2 (72 kDa) and TxAS (60 kDa) were measured by Western blot analysis, and GAPDH (36 kDa) was used as a loading control. Figure is the representative result selected from three independent experiments. Densitometry for blots was shown in the right panels. **p < 0.01 and ***p < 0.001, as compared to control; # *p* < 0.05 and ## *p* < 0.01 as compared with TNF-α treatment. **C.**, MTS assays were conducted to show the effects of GA on MTX cytotoxicity with regard to cell proliferation. There is a synergistic effect from the treatment with both GA and MTX on cell proliferation of RA FLS, suggesting GA could be used as an adjunctive agent not only to enhance the chemotherapeutic effects of MTX but also to reduce the negative side effects associated with MTX. Data are presented as percentages of the control and expressed as mean ± SD of three independent experiments done in triplicate. **p < 0.01 when compared to control. Abbreviations: COX-2, cyclooxygenase-2; GA, glycyrrhetinic acid; MTX, methotrexate; RA FLS, rheumatoid arthritis fibroblast-like synoviocytes; TxAS, thromboxane synthase.

Altogether, the possibility of that GL and GA are utilized as the useful additive to RA treatments has been well-studied in experiments and the underlying mechanisms is, at least in part, attributable to suppression of the COX-2/TxA2 pathway. The ongoing study in our laboratory has preliminarily supported this possibility (Figure 4), and the further studies in this field are expected.

## CONCLUSIONS

RA exerts profound influence on health-related quality of life, which imposed huge burdens on patients physically, mentally, and economically [63]. The general effectiveness of typically prescribed medications for patients with RA, including NSAIDs and DMARDs, has been far from satisfactory [1, 64]. Because COX-2-derived TxA2 is not the molecular target of NSAIDs and DMARDs, the limitations and negative side effects of those drugs may be, at least in part, attributable to lack of the effects on the COX-2/TxA2 pathway, which coupled with the positive role of the COX-2/TxA2 pathway in pathogenesis of RA suggests that the pharmacological approaches targeting this pathway hold the potential as a novel add-on therapy in therapeutic strategy of RA (Figure 3).

Understanding the mechanisms of action of the herbs may provide new treatment opportunities for RA patients, and the herbs used in traditional medicines provide a rich reservoir for extracting biologically active compounds. Licorice or the roasted licorice is one of the oldest and most frequently used botanicals in traditional Chinese medicine. This herb has been incorporated into recipes not only to enhance taste, but also to treat various conditions including inflammation [65]. GL and GA, active components of licorice, have been reported to target the COX-2/TxA2 pathway (Figure 3). Therefore, GL or GA could be used as an adjunctive agent in RA treatment not only to enhance the therapeutic effects of NSAIDs and DMARDs but also to reduce the adverse effects associated with NSAIDs and DMARDs (Figure 3). In a word, the COX-2/TxA2 pathway could be a crucial mechanism underlying the toxicity reducing and efficacy enhancing effects of GL and GA on typically prescribed medications, NSAIDs and DMARDs in principle, for treatments of patients with RA (Figure 3). Many researches have confirmed the toxicity reducing and efficacy enhancing effects of GL and GA on RA treatment [60-62], while further studies leading to the final application of this finding in the clinical management of RA are urgently required in the future.

## Abbreviations

ACTN1: α-actinin-1
bDMARDs: biologic disease-modifying anti-rheumatic drugs
cDMARDs: conventional disease-modifying anti-rheumatic drugs
COXIBs: selective COX-2 inhibitors
CREB: cAMP response element-binding protein
COX-2: cyclooxygenase-2
DMARDs: disease modifying anti-rheumatic drugs
ERK: extracellular signal-regulated kinas
GA: glycyrrhetinic acid
GL: glycyrrhizin
HMGB1: high mobility group protein B1
IL: interleukin
LEF: leflunomide
MAPKs: mitogenactivatedproteinkinases
MMPs: matrix metalloproteinase
MTX: methotrexate
NF: nuclear factor
NSAIDs: non-steroidal anti-inflammatory drugs
PGE2: prostaglandin E2
PGES: prostaglandin E synthase
PGH2: prostaglandin H2
PGI: prostacyclin
PGIS: prostacyclin synthase
PI3K: phosphoinositide-3-kinase
RA: rheumatoid arthritis
TNF: tumor necrosis factor
TP: thromboxane A2 receptor
TxA2: thromboxane A2;TxAS, thromboxane synthase
VEGF: vascular endothelial growth factor.
